# Multi-Scale Model for the Aging Performance of Particle-Filled Polymer Composites

**DOI:** 10.3390/polym15153158

**Published:** 2023-07-25

**Authors:** Congli Fang, Huizhen Wang, Yujiao Zhang, Minghua Zhang, Tao Shen, Jianke Du

**Affiliations:** Smart Materials and Advanced Structure Laboratory, School of Mechanical Engineering and Mechanics, Ningbo University, Ningbo 315211, China; 2011081025@nbu.edu.cn (C.F.); wwwhz777@163.com (H.W.); 2111081032@nbu.edu.cn (Y.Z.); zhangminghua@nbu.edu.cn (M.Z.)

**Keywords:** particle-filled polymer composite, accelerated aging, multi-scale model, thermal oxidative aging

## Abstract

In this study, we developed a novel multi-scale model to predict the aging performance of particle-filled polymer composites (PFPCs) under thermo-oxidative aging conditions. To investigate the aging behavior, high-temperature accelerated aging tests were conducted in combination with macroscopic and microscopic characterization. At the microscopic level, the crosslinking density of the polymer matrix is calculated using the closed-loop chain reaction of polymer oxidation. In addition, the theory of polymer physics was used to determine the relationship between crosslinking density and elastic modulus. Relationships between elastic modulus and dewetting strain were analyzed at the macroscopic level. Based on the observations and analyses, a multi-scale model was developed to predict the aging performance of PFPCs. The predicted results show good agreement with the test results, which verifies the reliability of the model.

## 1. Introduction

Particle-filled polymer composites (PFPCs) are a type of composite material that incorporates inorganic particles into a polymer matrix. These materials exhibit outstanding properties similar to those of pure polymers, including high specific strength, corrosion resistance, and elasticity, while also demonstrating good dimensional stability, creep resistance, and comprehensive mechanical properties [1,2]. However, for the long-term use of these materials, the degraded mechanical properties and failure due to thermo-oxidation should be considered, as they impact both the safety and cost-effectiveness, particularly for special functional materials like solid fuel used in aerospace applications [2,3,4,5]. Thus, it is of great significance to explore the mechanism of thermo-oxidative aging of PFPCs and establish an effective model to predict the aging performance.

Thermo-oxidative aging of the polymer is a set of irreversible chemical reactions, resulting in the changes of micro-scale molecular configuration and macro-scale mechanical properties [6]. As oxygen infuses into the polymer, oxidative crosslinking occurs, which leads to the increase in modulus and decrease in failure strain [7]. In the case of PFPCs, it is also reported that thermo-oxidative aging deteriorates the interface of the filler and polymer matrix [8,9,10]. Despite the extensive research on thermo-oxidative aging of polymers and PFPCs, it remains a challenging task to correlate aging characteristics at various scales, so as to establish an effective predictive model.

The Arrhenius law, combined with accelerated aging tests, are usually used to predict the thermal aging properties in polymers and PFPCs [11,12,13,14,15,16]. However, the Arrhenius law can be applied only to an elementary reactive process, while thermal aging of polymers includes at least six elementary reactions. A generalized Arrhenius law might not accurately describe the aging process [17,18,19,20,21,22]. Recently, attention has begun to focus on the closed-loop chain reaction of polymer oxidation and the corresponding multi-scale aging models [23]. However, related studies on PFPCs are still limited.

In this study, accelerated aging experiments are performed on the PFPCs, which consist of a matrix of hydroxyl-terminated polybutadiene/toluene diisocyanate and ammonium perchlorate particles. Crosslinking density, modulus, and debonding strain are evaluated after the aging experiments. The oxidation chain reaction rate, crosslinking density, and macro-scale mechanical properties are correlated to establish a multi-scale model with high reliability. This provides a novel reference framework for predicting the thermo-oxidative aging performance of PFPCs.

## 2. Materials and Methods

**Figure 1:**
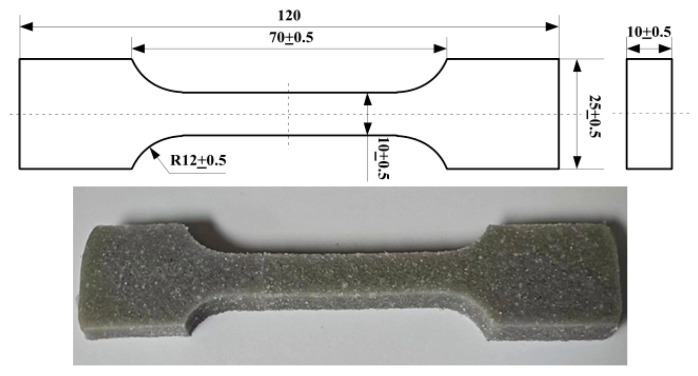
Diagram of specimen (Unit: mm).

In this study, we collaborated with the manufacturer to obtain a composite material consisting of a matrix of hydroxyl-terminated polybutadiene/toluene diisocyanate and ammonium perchlorate particles (with a diameter of 50–200 μm). The dimensions of the samples are shown in Figure 1. The filler-to-matrix mass ratio was 17:2. The composite samples were subjected to high-temperature accelerated aging tests.

In conducting aging tests, careful consideration must be given to the testing temperature and sampling time. The selection of the test temperature depends on the type of PFPCs used and the experimental objectives. However, when performing accelerated aging experiments, a conflict between theoretical and practical temperatures may arise. If the test temperature is too high, it can accelerate the aging process and shorten the testing time, but this may result in a significant deviation from the actual storage temperature and cannot accurately reflect the reaction process under real actual storage conditions. On the other hand, if the selected test temperature is too low, it will cause a slow aging process and a longer testing time, which is not consistent with the objective of accelerated aging. Therefore, the following principles should be followed when selecting temperature points for artificial accelerated aging experiments:

1. To ensure the accuracy of the experiment, the lowest aging temperature should be as close to the actual storage temperature of the propellant as possible, but not too close, to avoid excessively extending the testing time;
2. The highest aging temperature should be as large as possible to achieve the strongest accelerating effect while ensuring experiment safety and not changing the failure mechanism;
3. It is generally recommended to control the number of acceleration temperature points within three to five, which can balance the accuracy of data statistical analysis and the cost saving of experiments.

If the number of temperature points is too small, it may cause data deviation. Conversely, if the number of temperature points is too large, it will increase the cost of the experiment. According to these principles, this study selected three aging temperatures: 60, 70, and 80 °C to investigate the accelerated aging properties of PFPCs. Table 1 details the corresponding test conditions.

**Figure 2:**
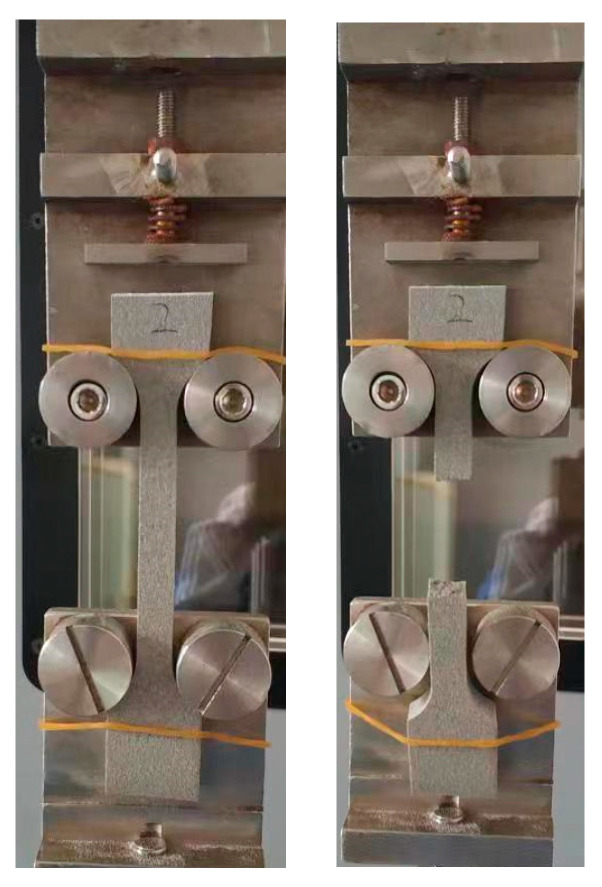
Diagram of specimen before and after tensile testing.

**Figure 3:**
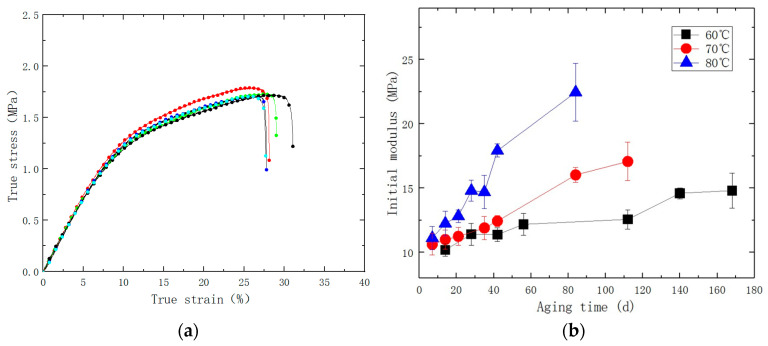
(**a**) Line chart of the relationship between true stress and true strain; (**b**) Line chart of the relationship between initial modulus and aging time during accelerated aging process.

The test temperature of uniaxial tensile testing is 25 ± 2 °C, and the relative humidity is not higher than 70%. Uniaxial tensile tests were conducted on the aged specimens at a testing speed of 100 mm/min to determine the corresponding initial modulus, which is presented in Figure 2 and Figure 3.

**Figure 4:**
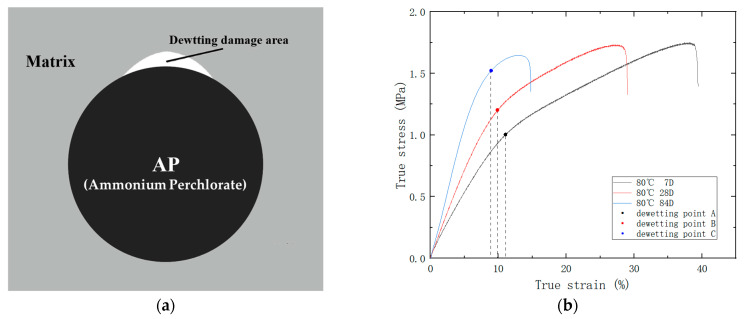
(**a**) Dehumidification schematic diagram of particle filled polymer composites; (**b**) Diagram of the dewetting point for particle-filled polymer composites.

**Figure 5:**
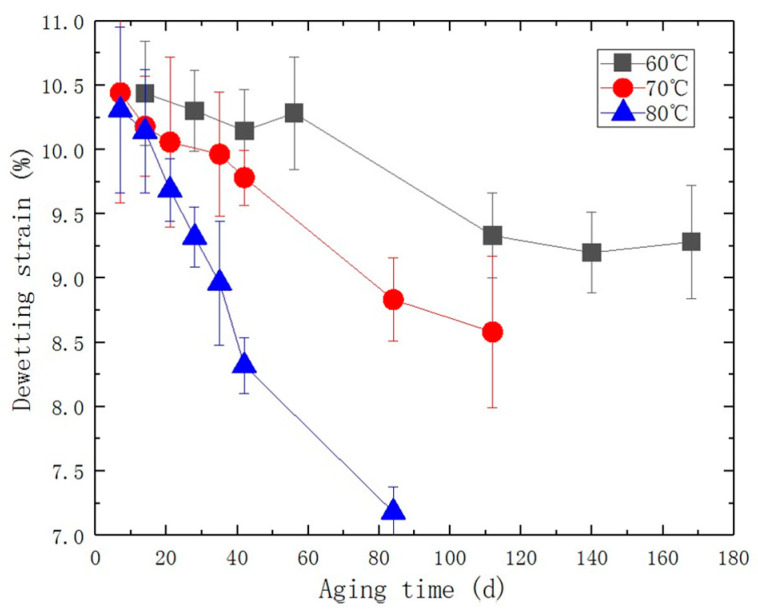
Line chart of the relationship between dewetting strain and aging time during accelerated aging process.

In this study, the maximum size of solid particles in the PFPCs can reach up to 200 μm. At this scale, it has been demonstrated that a significant amount of interfacial debonding occurs before matrix failure is shown in Figure 4a [24,25,26]. The stress-strain curve in Figure 3a clearly shows a noticeable change in slope when interfacial debonding takes place. This change indicates a decrease in the growth effect of stress with strain. Referred to as the “dewetting point,” it is the point at which the slope experiences the maximum change during the debonding process. To analyze the experimental data, equidistant strain form was used, where ε_k_ corresponds to the stress value σ_k_ = σ(ε_k_), with k = 0, 1, …, n. The first-order difference with a step size of Δε is represented by Δσ_k_ = σ_k+1_ – σ_k_, and the second-order difference is given by Δ^2^σ_k_ = Δσ_k+1_ – Δσ_k_. The “dewetting point” is determined at the point where the absolute value of Δ^2^σ_k_ reaches its maximum. The stress and strain at this point are referred to as the dewetting stress and dewetting strain, respectively. It should be noted that thermo-oxidative aging can cause a decline in bonding performance, leading to a reduction in the dewetting strain, as shown in Figure 4b. The dewetting strain of each aged specimen was determined based on the stress-strain curve. This data is presented in Figure 5.

With the initiation of PFPCs aging, the chemical structure of its polymer matrix undergoes certain changes (e.g., chain scission, chain crosslinking), which can be attributed to various environmental factors including oxidation and temperature. The crosslinking density serves as a critical indicator of the degree of crosslinking in polymers and is crucial for the performance of PFPCs. Specifically, the magnitude of crosslinking density affects not only the elastic properties of the polymer, such as initial modulus, fracture strength, maximum elongation, but also dynamic mechanical properties such as viscoelasticity. Thus, monitoring changes in crosslinking density provides an effective approach to investigate the alteration of polymer network structure during the aging process of PFPCs.

There are various methods available for measuring crosslinking density. In this study, the equilibrium swelling method was selected to determine the changes of crosslinking density in PFPC during the aging process. The basic principle of measuring crosslinking density of polymers by the swelling method is that the maximum swelling value of polymeric chains in the solvent is inversely proportional to the value of crosslinking density when the polymers reach swelling equilibrium. As the equilibrium swelling volume increases, the corresponding crosslinking density decreases; conversely, when the equilibrium swelling volume decreases, crosslinking density increases.

The specimens for the equilibrium swelling experiment were first cut into small pieces measuring 5 mm × 10 mm × 20 mm. The weight of each small piece was then measured using an electronic balance, and the weight data, M_0,_ was recorded. Subsequently, the weighed pieces were individually placed into 60 mL white wide-mouth bottles and a suitable amount of toluene solution (25~30 mL) was poured into each bottle, ensuring complete submersion of the specimen. Following this, the bottles were capped and left to soak for seven days, allowing for thorough swelling of the specimen. After this period, the specimens were gently removed using tweezers and placed on glass paper for flipping, and then the liquid on the glass paper was wiped off using filter paper. This flipping and wiping process was repeated until no more obvious liquid traces remained on the glass paper. Once this was done, the swollen specimens were swiftly transferred to pre-weighed vials with tight caps. The weight of the swollen specimens, M_1_, was then measured using an electronic balance. To complete the process, the accurately weighed swollen specimens were subjected to low-temperature, reduced-pressure drying in a vacuum drying oven until their weight no longer changed. Finally, the dried weight of the specimens, M_2_, was obtained by taking them out and precisely weighing them again.

We obtained experimental data through the equilibrium swelling experiment and calculated the swelling ratio, Q, based on Equation (1). Using the swelling equilibrium equation for cross-linked polymers (Equation (2)), we calculated the average molecular weight between crosslinking points, and further obtained the value of crosslink density according to Equation (3). Equation (1) is as follows,

(1) $$Q=\frac{\frac{M_{2}–\eta_{1}\;M_{0}}{\rho_{PH}}+\frac{M_{1}–{\;M}_{2}}{\rho_{1}}}{\frac{M_{2}–\eta_{1}\;M_{0}}{\rho_{PH}}},$$

where $\eta_{1}$ represents the mass percentage of AP particles in PFPCs; $\rho_{1}$ represents the density of toluene solvent; and $\rho_{PH}$ represents the density of the polymer matrix. Equation (2) is:

(2) $$\frac{{\bar{M}}_{c}}{\rho_{PH}\;{\tilde{V}}_{1}}\;(\;\frac{1}{2}\;–\;\chi_{1}\;)=Q^{\frac{5}{3}},$$

where $\chi_{1}$ represents the Huggins parameter; ${\tilde{V}}_{1}$ represents the molar volume of toluene solvent; and ${\bar{M}}_{c}$ represents the average molecular weight between crosslinking points in the sample. Finally, Equation (3) is defined as:

(3) $$\nu =\rho \;/{\bar{M}}_{c}.$$

**Figure 6:**
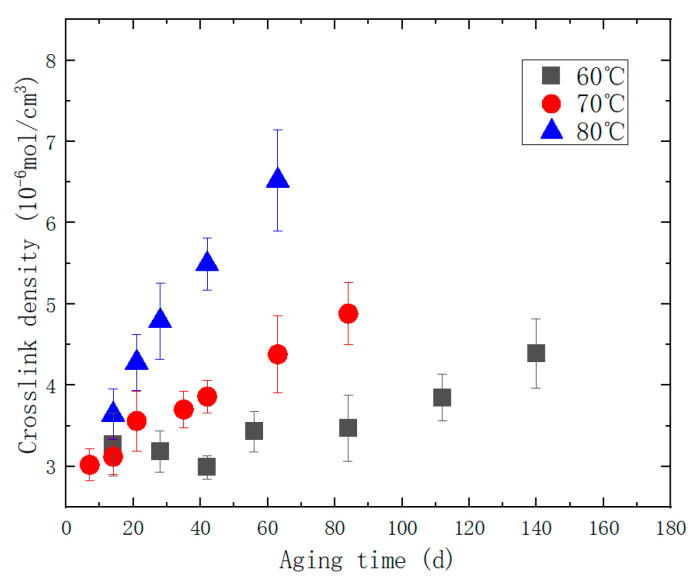
Scatter plot of the relationship between crosslink density and aging time during accelerated aging process.

The crosslink density of the aged samples obtained from the experiment is shown in Figure 6.

## 3. Microscopic Reaction Mechanism

### 3.1. Chain Reaction of Oxidation

Polymer matrix aging is a multifaceted chemical reaction process that involves numerous fundamental reactions at the molecular level. These reactions lead to alterations in the material’s physical properties. Six fundamental oxidation aging reactions involve bond-making and bond-breaking mechanisms [27]. These reactions are numbered as “I” through “VI” in Table 2.

### 3.2. Oxygen Consumed Rate 

Each reaction in this series has a corresponding Arrhenius equation that determines the rate constant, $k_{i}$, which is essentially a fundamental equation between the rate constant k_i_ and the absolute temperature, T:

(4) $$k_{i}=A\;exp\;\left[-E_{a}\;/\;\left(R\;T\right)\right],$$

where *A* represents the pre-exponential factor, *E_a_* represents the apparent activation energy, and R represents the ideal gas constant in this reaction.

Reaction (I) is called the Initiator of the oxidation reaction, as it tends to dominate the initiation stage. In the case of the propagation reaction, the rate constant $k_{II}$ for Reaction (II) is much faster than the rate constant $k_{III}$ for Reaction (III). Therefore, under conditions of excess oxygen availability, Reaction (III) becomes the dominant rate-controlling process. Additionally, the presence of excess oxygen results in the rapid transformation of alkyl radicals (P·) to peroxyl radicals (PO_2_·), with the termination reaction primarily occurring via Reaction (VI). Under the steady-state assumption, it was reported in the literature [23] that the overall oxidation rate in the case of excess oxygen can be linked to the constant value of $k_{1}=k_{III}\sqrt{k_{I}/2k_{VI}}$. Conversely, under conditions of limited oxygen availability, Reaction (II) becomes the dominant rate-controlling process. The limited oxygen leads to the accumulation of numerous alkyl free radicals (P·), unable to convert into peroxide radicals (PO_2_·).

As a result, the termination reaction mainly occurs via Reaction (IV), where the overall rate can be linked to the constant value of $k_{2}=k_{II}\sqrt{k_{I}/2k_{IV}}$.

To calculate the oxygen consumption rate, $S_{Ox}$, under different oxygen concentrations, the following hyperbolic model was selected, which can represent the oxygen consumption rate according to oxygen concentration:

(5) $$S_{Ox}=\frac{k_{1}{\;k}_{2}\;\left[O_{2}\right]\left[PH\right]}{k_{2}\;\left[O_{2}\right]+k_{1}\;\left[PH\right]}$$

where [O_2_] represents the concentration of oxygen in the specimen; and [PH] represents the concentration of polymer matrix in the specimen.

### 3.3. Concentration of $O_{2}$ and PH

There are two sources of oxygen: the oxygen dissolved in the matrix from the air and the oxygen generated by the decomposition of the ammonium perchlorate particles. However, the thermal decomposition of ammonium perchlorate requires a high temperature, which is difficult to achieve during actual storage. Therefore, we focus mainly on the calculation of oxygen carried by the air.

According to Henry’s law, the solubility of molecules such as oxygen in polymers is often low. The equation for the equilibrium oxygen concentration on the specimen surface, [O_2_]_s_, is shown in Equation (6):

(6) $${\left[O_{2}\right]}_{s}={Sol}_{O_{2}}\;\cdot \;p_{O_{2}},$$

where *Sol*_*O*_2__ represents the oxygen solubility coefficient in the adhesive. According to the van’t Hoff equation, it is related to the absolute temperature, T, as shown in Equation (7):

(7) $${Sol}_{O_{2}}={Sol}_{O_{2}0}\;\cdot \;exp\left[{-H}_{S_{O_{2}}}\;/\;\left(R\;T\right)\right],$$

where R represents the ideal gas constant and *Hs*_*O*_2__ represents the enthalpy value of *O*_2_ in the adhesive. 

The thickness of the specimens used in the accelerated aging experiments is relatively thin. Therefore, [O_2_]_s_, can be approximated by the oxygen concentration [O_2_] inside the specimen. 

The matrix concentration, [PH], of the polymer can be obtained by Equation (8):

(8) $$\left[PH\right]=\left(\;\rho_{PH}/M_{PH}\;\right)\;\left(\;V_{PH}\;/\;V\;\right),$$

where $\rho_{PH}$ represents the density of the polymer matrix; $M_{PH}$ represents the Molar mass of the polymer matrix; $V_{PH}$ represents the volume of the polymer matrix contained in the PFPC specimen; and *V* represents the total volume of PFPC specimens.

## 4. Construction of Multi-Scale Models

### 4.1. Relation between Reaction Rate and Crosslink Density

In this study, we do not consider the rate of polymer bond decomposition and the rate of oxidative crosslinking separately. Instead, we treat them as a whole, considering both as products of the oxidation reaction. Therefore, we focus only on the changes in crosslink density caused by the oxidation reaction. The evolution rate of the crosslink density (ν) was assumed to be directly proportional to the oxygen consumption rate of the matrix. Therefore, the crosslink density of the polymer is given by Equation (9):

(9) $$\nu =\nu_{0}+\omega \;S_{Ox}\;t,$$

where $\nu_{0}$ represents the initial crosslink density; *ω* represents the ratio of oxygen consumption rate to the rate of crosslink density change; *t* represents the aging time.

The function of crosslink density based on molecule concentration can be obtained from Equations (5) and (6). Substituting the rate constants, k_I_ and k_II_, into Equation (4), the predicted value of crosslink density can be expressed as Equation (10):

(10) $$\nu ={\;\nu }_{0}+\omega \;\frac{A_{1}\;e^{\frac{-E_{a1}}{R\;T}}\;\left[O_{2}\right]}{1+\frac{A_{1}\;e^{\frac{{-E}_{a1}}{R\;T}}\;\left[O_{2}\right]}{A_{2}\;e^{\frac{-E_{a2}}{R\;T}}\;\left[PH\right]}}\;t,$$

where *A*_1_, *A*_2_ represent the pre-exponential factor of k_I_, k_II_; *Ea*_1_, *Ea*_2_ represent the activation energy of k_I_, k_II_, respectively; R represents the ideal gas constant; T represents the absolute temperature; [O_2_] represents the concentration of oxygen in the specimen; and [PH] represents the concentration of polymer matrix in the specimen.

### 4.2. Relation between Crosslink Density and Modulus

According to the elasticity theory of cross-linked linear polymers [28], the network shear modulus, *G*, is proportional to the sum of the number densities of cross-links and entanglements, which act as effective cross-links. The Poisson’s ratio of the specimen is approximately 0.495, indicating that its elastic modulus, *E*, is also proportional to the sum of cross-links density and entanglements density as shown in Equation (11),

(11) $$E=\mu \;\left(\;\nu +1\;/\;N_{e}\;\right).$$

By combining Equations (10) and (11), a predictive model for the variation of elastic modulus of the specimen with aging temperature and aging time can be obtained, as shown in Equation (12):

(12) $$E=E_{0}+\mu \;\omega \;\frac{A_{1}\;e^{\frac{-E_{a1}}{R\;T}}\;\left[O_{2}\right]}{1+\frac{A_{1}\;e^{\frac{{-E}_{a1}}{R\;T}}\;\left[O_{2}\right]}{A_{2}\;e^{\frac{-E_{a2}}{R\;T}}\;\left[PH\right]}}\;t.$$

### 4.3. Relation between Modulus and Dewetting Strain

Numerous studies have demonstrated a statistical correlation between the elastic modulus after aging and the dewetting strain. According to the relevant research [23], a power function was employed to fit the mathematical relationship between dewetting strain and initial modulus.

The relationship between the dewetting strain, *ε_m_*, and the initial modulus, *E*, can be expressed as Equation (13):

(13) $$\varepsilon_{m}=\alpha \;E^{\beta }.$$

A complete multiscale model, represented by Equation (14), is obtained for the decay of dewetting strain with aging time, by combining the statistical model of dewetting strain with initial modulus, represented by Equation (13), with the predictive model for the variation of elastic modulus with aging temperature and aging time, represented by Equation (12):

(14) $$\varepsilon_{m}=\alpha \;{\left(\;E_{0}+\mu \;\omega \;\frac{A_{1}\;e^{\frac{-E_{a1}}{R\;T}}\;\left[O_{2}\right]}{1+\frac{A_{1}\;e^{\frac{{-E}_{a1}}{R\;T}}\;\left[O_{2}\right]}{A_{2}\;e^{\frac{-E_{a2}}{R\;T}}\;\left[PH\right]}}\;t\;\right)}^{\beta }.$$

## 5. Determination of Parameters in Multi-Scale Model

### 5.1. Parameters for Calculation of Crosslink Density

By substituting the molecular concentration into Equation (10), a predictive model for the crosslink density of the specimen can be further derived as a function of aging temperature and aging time, as shown in Equation (15):

(15) $$\nu =\nu_{0}+\omega \;\frac{A_{1}\times e^{\frac{-E_{a1}}{R\;T}}\times {Sol}_{O_{2}0}\times {\;e}^{\frac{-H_{sO_{2}}}{R\;T}}\times {\;P}_{O_{2}}}{1+\frac{A_{1}\times {\;e}^{\frac{-E_{a1}}{RT}}\times {Sol}_{O_{2}0}\times e^{\frac{-H_{sO_{2}}}{R\;T}}\times {\;P}_{O_{2}}}{A_{2}\times e^{\frac{-E_{a2}}{R\;T}}\times \left({\;\rho }_{PH}/M_{PH}\;\right)\;\left(\;V_{PH}\;/\;V\;\right)}}\times t.$$

The parameter values of ${Sol}_{O_{2}0}$, $H_{sO_{2}}$, *Ea*_1_, and *Ea*_2_ can be found by referring to relevant literature [29,30].

**Figure 7:**
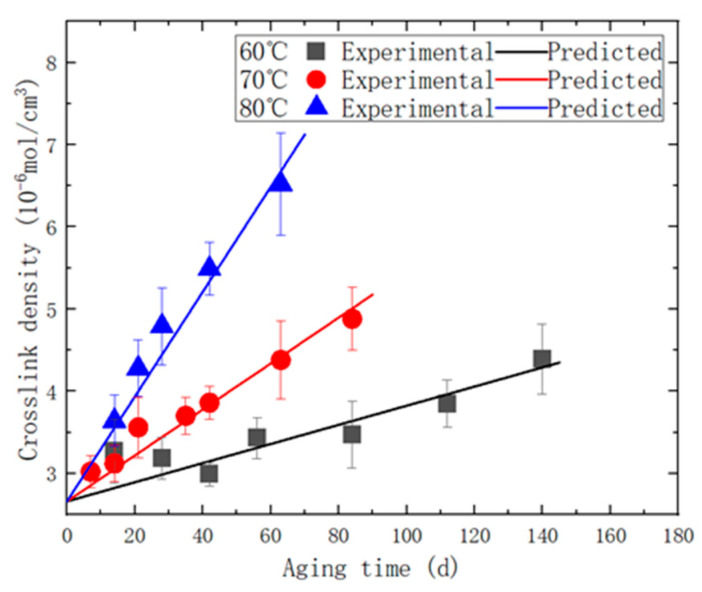
The comparative diagram of experimental/predicted crosslink density.

We organized the crosslinking densities of the specimens aged at 60, 70, and 80 °C, which were measured using the equilibrium swelling method. Subsequently, we performed fitting by substituting these values into Equation (15). This analysis allowed us to obtain the pre-exponential factors, *A*_1_ and *A*_2_, as well as the ratio, *ω*, between the rate of oxygen consumption and the rate of change in crosslinking density (Figure 7).

### 5.2. Parameters for Calculation of Initial Modulus

**Figure 8:**
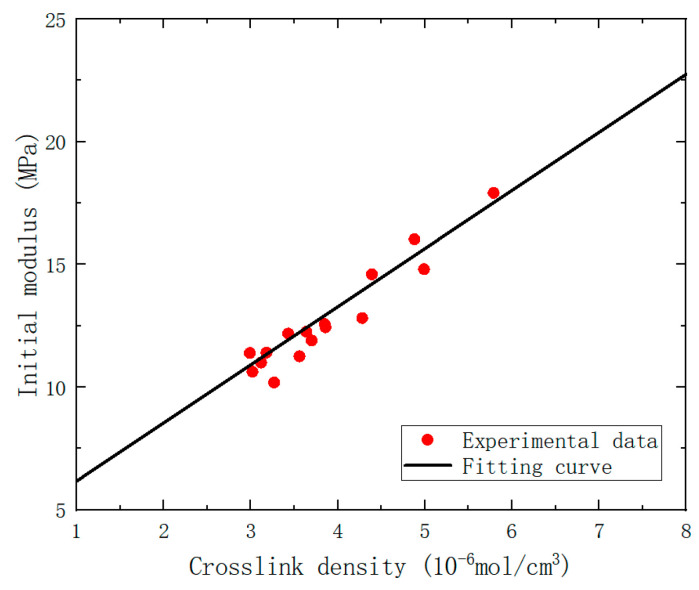
Diagram of crosslink density-initial modulus fitting for the specimen.

The crosslink density data obtained from equilibrium swelling experiment and the initial modulus data obtained from uniaxial tensile tests were processed and fitted using a linear function to obtain the values of μ, as shown in Figure 8.

### 5.3. Parameters for Calculation of Dewetting Strain


(16) $$\varepsilon_{m}=\alpha \;E^{\beta }=\alpha \;{\left(\;\mu \;\left(\;\nu +\;1\;/{\;N}_{e}\;\right)\;\right)}^{\beta }$$

**Figure 9:**
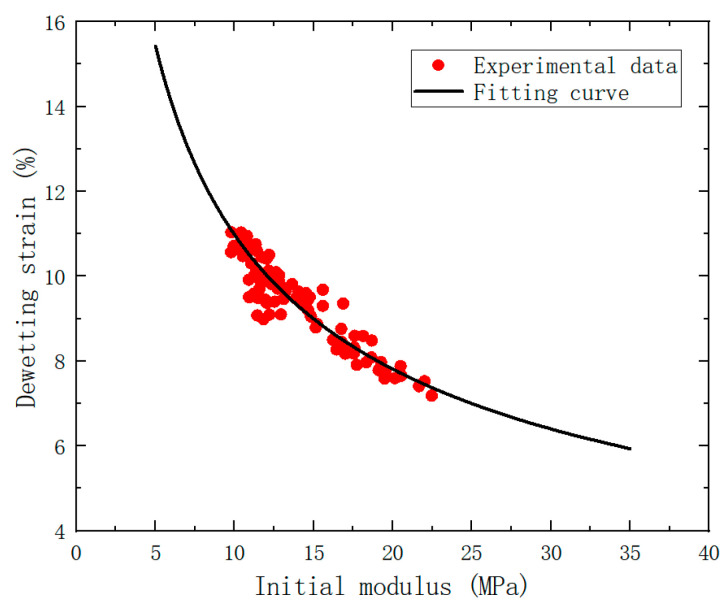
Diagram of initial modulus-dewetting strain fitting for the specimen.

The initial modulus and dewetting strain data obtained from uniaxial tensile tests were processed and fitted using a power function to obtain the values of *α* and *β* (Figure 9). The results were compiled and tabulated in Table 3.

### 5.4. Reliability Analysis

**Figure 10:**
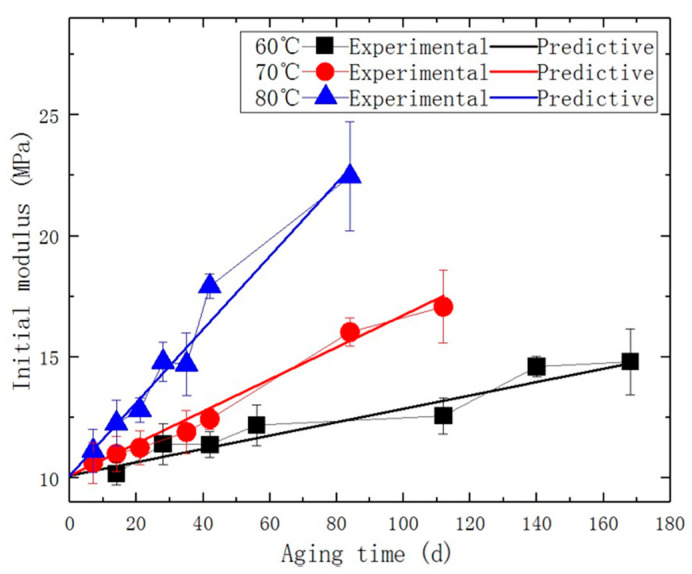
The comparative diagram of experimental/predicted initial modulus density.

By substituting the parameters in Table 3 into Equation (9), a predictive model for the initial modulus of the specimen, as a function of aging temperature and time, was obtained, as shown in Figure 10 and Equation (17), which is:

(17) $$E=10.1+2.37\times 10^{6}\times \left(\;43.64\times \frac{6.4\times e^{\frac{-6527}{T}}}{1+1.8{\times 10^{-3}\times e}^{\frac{3532}{T}}}\times t\;\right).$$

By comparing the predicted values of initial modulus with the measured values obtained from the uniaxial tension test, it is found that both exhibit a similar increasing trend. The predicted growth rate at different temperatures is highly consistent with the experimental results. The value of R-square is 0.936, indicating that the prediction model has a high degree of accuracy and reliability.

By substituting the parameters in Table 3 into Equation (14), a predictive model for the dewetting strain of the specimen, as a function of aging temperature and time, is obtained, as shown in Equation (18):

(18) $$\varepsilon_{m}=34\times {\left(\;10.1+43.64\times \frac{1.52\times 10^{7}\times {\;e}^{\frac{-6527}{T}}}{1+1.8{\times 10^{-3}\times e}^{\frac{3532}{T}}}\times t\;\right)}^{-0.493}.$$

**Figure 11:**
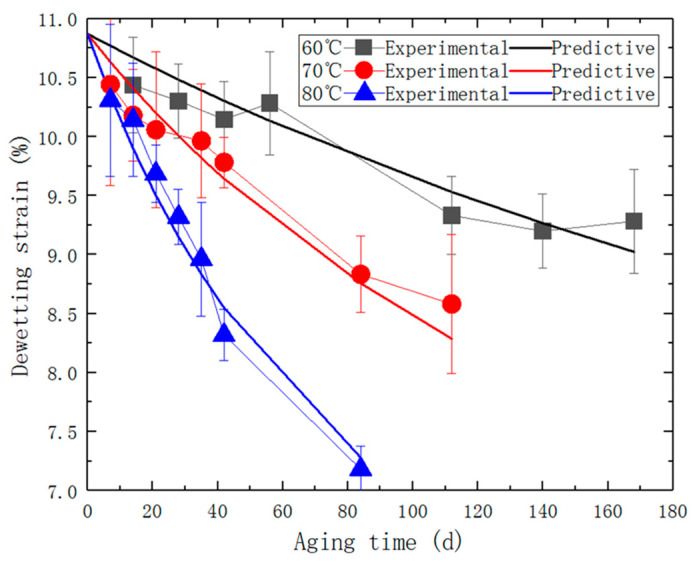
The comparative diagram of experimental/predicted dewetting strain.

Figure 11 displays the comparison of experimental/predicted dewetting strain.

It can be observed that the predicted dewetting strain follows a similar trend with the experimentally obtained data. The R-squared value for this predictive model is 0.929, indicating an acceptable accuracy and reliability.

In conclusion, we have established a correlation model from the small molecular scale to the large molecular scale by considering the linear relationship between oxygen consumption rate and cross-linking density. Based on polymer theory, we related the cross-linking density to the Young’s modulus, thus constructing a correlation model from the large molecular scale to the macroscopic scale. Finally, we have used statistical analysis to establish the mathematical relationship between initial modulus and dewetting strain, thereby linking the macroscopic scale to ageing characterisation. By integrating these models at different scales, we have successfully developed a multi-scale model. The model accurately predicts the evolution of initial modulus, dewetting strain over time, providing an effective method for characterizing the aging properties of this type of material.

With this model, we can more accurately estimate the ageing performance and life of PFPCs based on factors such as matrix concentration, ambient temperature, and storage time. This allows us to reduce risk and minimize waste. However, this model does not take into account certain ageing factors such as external stresses and temperature variations. Moreover, it simplifies the calculation of parameters such as oxygen concentration. There is many limitations and much room for improvement in these areas. Future research can focus on addressing these limitations and promoting the development of a more scientifically comprehensive multi-scale correlation model for PFPCs. This will allow for more accurate ageing predictions in the future.

## 6. Conclusions

In this study, an accelerated aging experiment was conducted on PFPC specimens to analyze the micro-reaction mechanism and macro-aging performance evolution during thermal oxygen aging. The relationship between oxygen consumption rate, crosslinking density, initial modulus, and dewetting strain was established based on the thermo-oxidative aging mechanism. Crosslinking density was measured using the equilibrium swelling method, while uniaxial tensile tests were performed to evaluate macro-aging performance, such as modulus and dewetting strain. Subsequently, a multi-scale correlation model, spanning from small molecular scale to large molecular scale to macroscopic scale, was constructed.

(1). We analyzed the micro-reaction mechanism during the thermal aging process of the samples and determined the relationship between oxygen consumption rate and crosslinking density. Correlation models were established at different scales, including the relationship between crosslinking density and Young’s modulus at the large molecular scale and a correlation model from the small molecular scale to the large molecular scale. Additionally, we statistically correlated the mathematical relationship between initial modulus and dewetting strain.

(2). A multi-scale model for PFPCs was developed, incorporating parameters from related work and utilizing calculation and parameter-fitting based on test data. 

(3). The predicted values from the multi-scale correlation model showed a high degree of consistency with experimental measurements, confirming the reliability of the model. This achievement enables multi-scale characterization of PFPC aging performance and provides a novel reference framework for predicting the thermo-oxidative aging performance of PFPCs.

## Figures and Tables

**Table 1:** Table of accelerated aging test conditions.

Temperature (°C)	Aging Time (d)							
60	14	28	42	56	84	112	140	168
70	7	14	21	35	42	63	84	112
80	7	14	21	28	35	42	63	84

**Table 2:** Table of simplified oxidation mechansm for polymer matrix.

Reaction Stage		Reaction Equation	Rate Constant
Initiation	I	$2\;\mathrm{POOH}\;\to \;PO_{2}\cdot +\;P\cdot +\;H_{2}O+\;\gamma P=O+\;(1-\gamma )\;\mathrm{POH}$	k_I_
Propagation	II	${P\cdot +\;O}_{2}\;\to \;{\mathrm{PO}}_{2}\cdot$	k_II_
	III	${\mathrm{PO}}_{2}\cdot +\;\mathrm{PH}\;\to \;\mathrm{POOH}+\;P\cdot$	k_III_
	IV	P·+ P·→ P-P	k_IV_
Termination	V	$P\cdot +\;{\mathrm{PO}}_{2}\cdot \to \;\;2\;\mathrm{PO}\cdot$	k_V_
	VI	${\mathrm{PO}}_{2}\cdot +\;{\mathrm{PO}}_{2}\cdot \to \;\mathrm{POOP}+\;O_{2}$	k_VI_

where PH represents polymer matrix; P· represents alkyl free radical; POOH represents peroxyhydrogen radical group; PO_2_· represents peroxyl radical; POOP represents inactive carbonyl product; and O_2_ represents oxygen.

**Table 3:** Table of the parameters in the formula.

Parameter	Unit	Value
$\nu_{0}$	mol·cm^−3^	$2.66\times 10^{-6}$
$A_{1}$	s^−1^	$1.94\times 10^{8}$
$A_{2}$	s^−1^	$7.33\times 10^{7}$
$E_{a1}$	J·mol^−1^	$5.424\times 10^{4}\;$[29]
$E_{a2}$	J·mol^−1^	$1.13\times 10^{5}\;$[29]
${Sol}_{O_{2}0}$	mol·cm^−3^·Pa^−1^	$1.55\times 10^{-12}$[30]
$H_{sO_{2}}$	J·mol^−1^	$1.2\times 10^{3}\;$[30]
ω	-	43.64
$E_{0}$	MPa	10.1
μ	MPa·mol^−1^·cm^3^	2.37$\;\times \;10^{6}$
α	-	34
β	-	−0.493
$\rho_{PH}$	g·cm^−3^	0.913
$M_{PH}$	g·mol^−1^	3$\;\times \;10^{3}$
$V_{PH}/V$	-	0.1594

## Data Availability

The raw/processed data required to reproduce these findings cannot be shared at this time as the data also forms part of an ongoing study.
